# The Efficacy and Success Rate of Methotrexate in the Management of Ectopic Pregnancy

**DOI:** 10.7759/cureus.26737

**Published:** 2022-07-11

**Authors:** Asma Khalil, Arwa Saber, Khulud Aljohani, Mohammad Khan

**Affiliations:** 1 Obstetrics and Gynecology, Ministry of the National Guard-Health Affairs, King Abdulaziz Medical City, Jeddah, SAU; 2 Medicine and Surgery, King Saud Bin Abdulaziz University for Health Sciences College of Medicine, Jeddah, SAU; 3 Medicine, King Saud Bin Abdulaziz University for Health Sciences College of Medicine, Jeddah, SAU

**Keywords:** extra-uterine pregnancy, pregnancy, single dose, methotrexate, ectopic pregnancy

## Abstract

Background

Ectopic pregnancy is a life-threatening medical condition wherein pregnancy occurs outside the uterus. This study primarily aimed to evaluate the efficacy of methotrexate (MTX) in treating patients diagnosed with early ectopic pregnancy between 2016 and 2021. Second, it aimed to investigate the clinical outcomes of subsequent pregnancies following ectopic pregnancy treatment.

Methods

A retrospective cohort study was carried out at a tertiary care centre in Jeddah, Saudi Arabia. The sample for this study comprised 59 patients who were diagnosed with ectopic pregnancy and treated with MTX at King Abdulaziz Medical City, Jeddah, between 2016 and 2021. The data were stored in the Microsoft Office Excel format and analyzed using the SPSS software.

Results

There were 10 reported cases of ruptured ectopic pregnancy; seven of these underwent surgery, while three were successfully treated conservatively using MTX. Success rates of 55.9% and 93.8% were recorded after administering the first and second dose of MTX, respectively, based on beta-human chorionic gonadotrophin (β-hCG) drop between day 4 and day 7. Among those treated with MTX, 37.3% of the patients reported a successful subsequent pregnancy, of which 54.5% delivered a live, healthy infant and 13.6% had a second ectopic pregnancy.

Conclusion

MTX proved effective in treating ectopic pregnancy, with a β-hCG drop of more than 15%. Patients who received a second dose of MTX showed a higher drop in β-hCG level, indicating greater success as a cumulative effect. The clinical outcome after MTX treatment of ectopic pregnancy was shown to be significant with a p-value <0.05.

## Introduction

An ectopic pregnancy occurs when a zygote implants outside the uterus [1]. An ectopic fertilized egg may be implanted in different sites such as the abdomen, ovaries, and broad ligaments. However, 98% of ectopic pregnancies occur in the fallopian tubes [2]. In 1990, CDC estimated that ectopic pregnancy accounts for 2% of all pregnancies [3]. Furthermore, the rate increased from 6% to 16% in women who presented to the ED complaining of vaginal bleeding and/or abdominal pain [3]. Moreover, a study conducted at the Royal Commission Medical Center (RCMC), Yanbu Industrial City, estimated that the rate of ectopic pregnancy was 0.58% at the center [4]. Further, a study conducted at King Fahd University Hospital, Al Khobar, to estimate the prevalence of ectopic pregnancy between January 1, 2000, and December 31, 2011, established that out of 24,098 deliveries, 274 (1.19%) were diagnosed as ectopic pregnancies [5]. The study concluded that the majority of cases (61.1%) involved women under the age of 30.

Ectopic pregnancy is considered a life-threatening condition which requires immediate intervention. Owing to the advancements in medical technology, ectopic pregnancy can now be diagnosed in the early stages. As a result, it can now be treated with medical modalities instead of surgical interventions. In this context, methotrexate (MTX), an antimetabolite used to treat certain neoplastic diseases, severe psoriasis, and adult rheumatoid arthritis, has been widely used as a medical approach to terminating an ectopic pregnancy. However, due to its toxicity, MTX is contraindicated in patients with impaired renal function, ascites, or pleural effusion [6].

In stable cases with unruptured ectopic pregnancy and low levels of beta-human chorionic gonadotrophin (β-hCG), the use of MTX has been associated with better outcomes [7]. A retrospective study was conducted in Shaanxi Provincial People’s Hospital, China, in 2020 [7]; the study comprised 238 women who were diagnosed with an ectopic tubal pregnancy and were administered a single dose of MTX. The β-hCG levels were measured on the initial, fourth, and seventh days of MTX administration. The clinical outcome was analyzed based on the complete resolution of β-hCG levels without needing an additional dose or a surgical intervention. The overall success rate of MTX therapy was found to be 69.75%. In addition, among the cases of failure, treatment with the second dose of MTX proved successful in 13.25% of patients. However, 30.25% of patients required further interventions. Moreover, the initial level of β-hCG was found to be a strong indicator of the success rates of MTX. Accordingly, patients with an initial β-hCG level >4000 IU/L had significantly higher success rates. Locally, 225 patients who received MTX as a primary intervention were reviewed as part of a study conducted at King Abdulaziz Medical City (KAMC), Saudi Arabia, in 2005 [1]. A single dose of MTX was found to be successful in 72% of the patients. However, 28% of the patients reported failure of the first dose of MTX. These cases required either a second dose of MTX (63%) or surgical intervention (37%). On the other hand, interventions for conception following ectopic pregnancy were associated with negative outcomes, such as post-ectopic pregnancy subfertility. Another study was conducted on 173 post-ectopic women who tried conceiving [8]. It was found that regardless of the intervention technique, 10 (5.8%) women were referred to an assisted reproductive technique to aid conception; among these, seven conceived successfully.

While the literature has reported varying success rates of MTX, few studies discuss the outcomes after treatment with MTX. Hence, there is a gap in the knowledge base, and the safety and efficacy of MTX are yet to be investigated. Therefore, this study aimed to evaluate the success rate of MTX in treating patients diagnosed with early ectopic pregnancy. This study also investigates the clinical outcomes following ectopic pregnancy with treatment with MTX.

## Materials and methods

Design

This study involved a retrospective analysis of all the patients with ectopic pregnancy who had been treated with MTX. The study included all health records from January 2016 to December 2021. In addition, the study utilized the files and documentation of the hospital staff and the ultrasound and laboratory reports to investigate the success rate of MTX in treating tubal ectopic pregnancies. The study was approved by the Institutional Review Board (IRB).

Area and population

The study was conducted in the obstetrics and gynaecology department of KAMC, Jeddah, Saudi Arabia. KAMC is a tertiary care centre with a bed capacity of 751 that serves all national guards’ affiliates in the western region of Saudi Arabia. The study population comprised women of reproductive age who were diagnosed with ectopic pregnancy and treated with MTX between 2016 and 2021.

Inclusion and exclusion

In this study, no sample size calculation or sampling techniques were used. As this was a hospital-based study, we aimed to include all the cases after applying the eligibility criteria.

Data collection

The data were obtained from the electronic health system through inspection of the patients' records, using a data collection sheet developed by the authors. The data collection sheet included all the variables of interest essential to achieving the study's objectives: sociodemographic characteristics, comorbidities, obstetric history, β-hCG levels, and sac size upon ultrasound.

Data analysis

Microsoft Office Excel was used for data entry. The SPSS Statistics for Windows version 25.0 (SPSS Inc., Chicago, Ill., USA) was used for the data analysis. The categorical and demographic variables were represented as frequencies and percentages, while continuous variables were represented as means and SDs. The independent student's t-test and Mann-Whitney test were conducted for continuous dependent variables. The Fisher's exact test was used for categorical dependent variables to determine the associated factors. P-values <0.05 were considered to be statistically significant.

## Results

A total of 59 patients diagnosed with ectopic pregnancy from 2016 to 2021 were included in this study. The mean age of the patients was 34.7 ± 4.2 years. The mean BMI was 29.9 ± 7.9 (Table 1). Ectopic pregnancy was diagnosed based on the ultrasound finding, which was correlated with the β-hCG level and sac size. In this study, four patients (6.8%) had a sac size of more than 5 cm, with three of those patients developing ruptured ectopic pregnancy.

**Table 1:** Demographic Data

	N	Mean	Std. Deviation
Age	59	34.7	4.2
Weight	59	71.4	15.3
Height	59	156.5	5.7
Body Mass Index (BMI)	59	29.9	7.9

Furthermore, all of the 59 patients had a tubal implemented ectopic pregnancy. After reviewing each patient's medical history, 12 out of 59 patients were found to have a previous history of ectopic pregnancy. The details about their medical history of ectopic pregnancy, tubal surgery, and assisted conception are shown in Table 2.

**Table 2:** The Patient's Medical History

	N=59	%
Previous Ectopic Pregnancy	12	20%
Tubal Surgery	7	11%
Assisted Conception	5	8%

**Figure 1:**
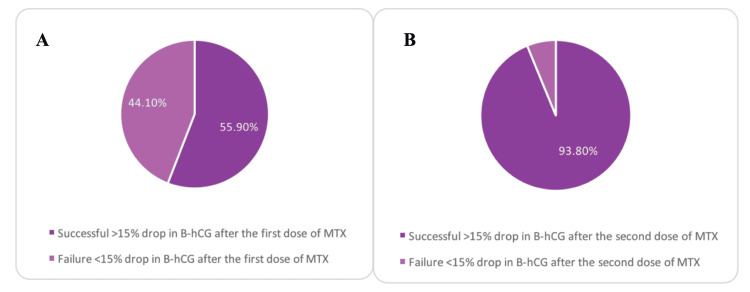
The success rate of the first (A) and second (B) dose of methotrexate. β-hCG: Beta-human chorionic gonadotrophin; MTX: Methotrexate.

The success of MTX is defined as the decrease in β-hCG level by more than 15% between four and seven days after treatment with MTX. Thus, from Figure 1A, it can be inferred that in 33 out of 59 (55.9%) patients, a single dose of MTX (1.0 mg/kg or 50 mg/m2 intramuscular without folinic acid) was sufficient to terminate the ectopic pregnancy. After administering the second dose of MTX, 93.8% of the patients had a successful termination of the ectopic pregnancy (Figure 1B). Among those, 7% (2/26) had a ruptured ectopic pregnancy that required surgical intervention, while 93% (24/26) of the patients had a plateau of β-hCG levels after the first dose of MTX. Only 10 out of 59 (16.9%) were diagnosed with a ruptured ectopic pregnancy. Among those who had ruptured ectopic pregnancies, seven patients required surgical intervention. Thus, 30% of patients with ruptured ectopic pregnancy were managed conservatively with MTX.

**Figure 2:**
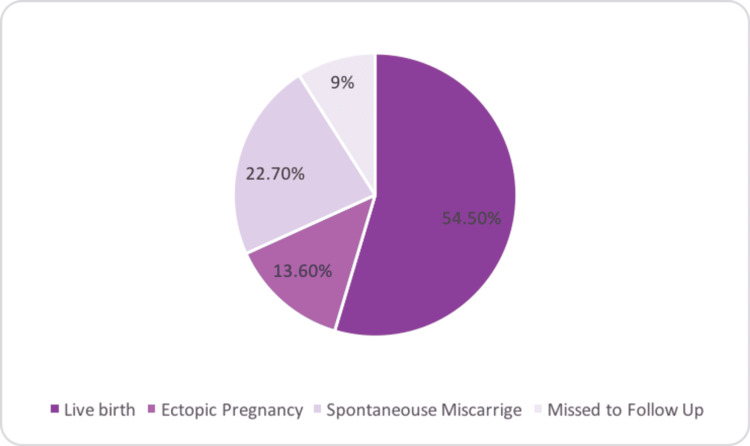
The fertility outcome of post methotrexate pregnancy.

In the follow-up after the MTX treatment of ectopic pregnancy, 22 out of 59 (37.3%) patients reported a successful pregnancy. Among the 59 patients, more than half (54.5%) delivered a live, healthy infant, while 13.6% had a second ectopic pregnancy. Moreover, 22.7% of the patients had a spontaneous miscarriage (Figure 2).

## Discussion

An ectopic pregnancy occurs when an embryo fails to implant within the uterine cavity. It is considered to be a life-threatening medical condition and can significantly impact morbidity and mortality [1]. Therefore, appropriate critical management is required in such conditions. The management may be classified as medical or surgical, depending on certain criteria. As part of the medical management, MTX may be given in single or multiple doses. It is indicated in patients who are hemodynamically stable and have an unruptured ectopic pregnancy, an initial low β-hCG level (≤10,000 mIU/mL), an adnexal mass with a diameter ≤ 5 cm, and amenorrhea [9]. On the other hand, absolute contraindications include chronic conditions such as chronic liver disease, pre-existing blood dyscrasias, pulmonary disease, peptic ulcer disease, and immunodeficiency [9].

This study demonstrated the success rate of MTX in treating ectopic pregnancy, which was correlated with the level of subsequent decrease in β-hCG levels and was considered successful if there was a >15% drop. The results of this study showed that after the first dose of MTX, 33 out of 59 (55.9%) patients showed a >15% drop in β-hCG levels on days 4 and 7 (Figure 1A). Similarly, a previous study reported a success rate of 65% with a single dose of MTX [10]. Moreover, 93.8% showed a >15% drop in β-hCG levels among the patients who received a second dose. Hence, patients who received a second dose of MTX showed superior results to those who received only one dose. This suggests that adequate follow-up is essential in individualized cases to determine the need for a second dose. In addition, the same study calculated the average time of resolution of ectopic pregnancy as 32 days for a single dose and 58 days for a second or third dose [10]. This can guide the management of ectopic pregnancy, help determine the prognosis, and answer patients' questions.

The mean gestational age was seven weeks at diagnosis, which is consistent with other studies, as ectopic pregnancy is usually diagnosed in the first trimester between 6 and 10 weeks [11]. The mean sac size on ultrasound was 2.25 cm, while another study showed that ≤5 cm is considered a good prognostic factor and associated with better clinical outcomes [12]. Four patients had a sac size above 5 cm, three of which had a ruptured ectopic pregnancy and subsequently required surgical intervention. This suggests that frequent sac size monitoring during an ultrasound can be used as a prognostic factor to check for the risk of complications such as ruptured ectopic pregnancy or the need for surgery. Among the 10 cases of ruptured ectopic pregnancy, seven underwent surgery, while three were stable and managed successfully with MTX. This suggests that MTX can be considered as a treatment for cases of ruptured ectopic pregnancy. A careful administration of MTX doses can greatly reduce the use of invasive procedures in ectopic pregnancy cases. In the current study, 10 patients underwent surgery, six of them due to ectopic pregnancy and others due to failure of medical treatment, which may have occurred due to other hidden factors.

The study sample had a mean age of 34 years. Now, advanced maternal age is considered a risk factor for ectopic pregnancy [13]. Furthermore, the risk of infertility after an ectopic pregnancy is greater among women older than 35 years [14]. Thus, advanced maternal age in this study may have affected the ectopic pregnancy risk and fertility. Moreover, although the BMI mean was 29.9 kg/m2 in this study, previous studies have reported an association between low BMI and ectopic pregnancy [15]. Therefore, this can indicate that abnormally high or low BMI is considered a risk factor for ectopic pregnancy.

Other factors considered were age at presentation, gravida, parity, history of abortion/miscarriage, previous ectopic pregnancy, tubal surgery, assisted conception, previous membrane rupture, complications, and surgery. No association was found between the aforementioned factors and patients with successful reduction in β-hCG after MTX treatment. Furthermore, 45% of patients never had an abortion or a miscarriage. In addition, although all patients were found negative for smoking and pelvic inflammatory disease (PID), research has proven that patients with PID have a 2.12 times higher risk of developing ectopic pregnancy [16]. However, ectopic pregnancy can occur spontaneously, and the presence of risk factors is not essential for diagnosis. In contrast, a previous ectopic pregnancy is a major risk factor for its recurrence [17]. This is in line with our findings, which showed that 20% of patients had a previous ectopic pregnancy. Moreover, 11.9% had undergone tubal surgery, which has also been associated with ectopic pregnancy [17]. Additionally, previously assisted conception was found among five patients (8.5%); this has also been shown to increase the risk of ectopic pregnancy by 0.8% to 8.6% compared to the general population [18]. Other reported risk factors for ectopic pregnancy include tubal damage caused by infection or surgery, smoking, and assisted reproduction [15].

The clinical outcomes following an ectopic pregnancy treated with MTX were evaluated as indicators of the ability of conception. Over one-third of the patients (37.3%) retained their fertility and had subsequent pregnancies. The outcome of the pregnancy was also determined and was shown to be statistically significant (p-value <0.05): 12 patients had live births, three had ectopic pregnancies, five had miscarriages, and two had a loss of follow-up.

This study provides supportive evidence of the high success rates of MTX for the medical treatment of ectopic pregnancies. However, subsequent infertility is still a major complication associated with the condition and its MTX treatment. The limitations of this study include a small sample size, which contributes to the low incidence of ectopic pregnancy. Conducting multi-centre studies may increase the sample size and enhance the outcomes of this research.

## Conclusions

In summary, the use of MTX, whether in a single dose or multiple doses, has been proven to be an effective alternative for the management of ectopic pregnancies and offers several benefits over invasive surgical treatment. The use of MTX is less invasive, is less expensive, can be given on an outpatient basis, and does not need expertise. Moreover, future fertility expectations are better managed with MTX, leading to higher intrauterine pregnancy and lower ectopic pregnancy rates. Therefore, the promotion of the use of MTX is highly recommended for the management of ectopic pregnancy in order to preserve fertility and avoid complications. Nevertheless, further studies are recommended to investigate the long-term effects of MTX.
